# From emotion regulation to suicide-specific coping: a qualitative study of self-regulatory processes in suicidal crises

**DOI:** 10.3389/fpsyt.2026.1858456

**Published:** 2026-07-24

**Authors:** Juliane Brüdern, Johanna Panitz, Lena Spangenberg, Katarina Stengler, Maria Strauss, Heide Glaesmer

**Affiliations:** 1 Department of Medical Psychology and Medical Sociology, University of Leipzig, Leipzig, Germany; 2 SRH University of Applied Sciences Heidelberg, Campus Gera, Gera, Germany; 3 Department of Psychiatry, Psychotherapy and Psychosomatics, Helios Park Hospital Leipzig, Leipzig, Germany; 4 Department of Psychiatry and Psychotherapy, University of Leipzig, Leipzig, Germany

**Keywords:** coping, emotion regulation (ER), qualitative research, self-control, suicidal thoughts and behavior (STB), self-regulation, suicidal ideation

## Abstract

**Introduction:**

Negative affect is strongly associated with suicidal thoughts and behaviors (STBs). Accordingly, the ability to regulate intense negative affective states may be central to understanding the emergence and maintenance of STBs. However, very little is known about how individuals with lived experience regulate negative affect and cope with suicidal thoughts and urges. This study aimed to explore emotion-regulation strategies (ERS) and suicide-specific coping strategies (SCS) used during suicidal crises, their perceived effectiveness, and the situational and contextual factors influencing their selection.

**Methods:**

Semi-structured interviews were conducted with 12 individuals admitted to a psychiatric hospital due to an acute suicidal crisis. Data were analyzed using qualitative content analysis.

**Results:**

Before and during suicidal crises, participants predominantly used avoidance-oriented ERS that contributed to the maintenance of negative affect. With increasing distress, suicidal thoughts emerged as an additional ERS, providing short-term relief while contributing to the intensification of the suicidal crisis over time. A broad range of SCS was identified, differing in their motivational orientation toward suicidal behavior. Suicide-approach strategies (e.g., suppression, substance use, preparatory behaviors) often facilitated the escalation of the suicidal crisis, whereas suicide-avoidant strategies (e.g., seeking support, safety strategies) were context-dependent and not consistently effective. Strategy selection and effectiveness were shaped by situational and contextual factors.

**Conclusion:**

Suicidal crises can be understood as dynamic, context-dependent self-regulatory processes characterized by escalating emotional distress, changes in strategy use, and dynamic regulatory resources. Interventions should focus on strengthening flexible coping repertoires and preserving self-regulatory capacity. Future research should further investigate these processes to improve prevention and intervention efforts.

## 1 Introduction

Suicidal thoughts and behaviors (STBs) constitute a major public health concern, with more than 700,000 people dying by suicide every year and high lifetime prevalence rates of suicidal thoughts in the general population (15.5% in Germany (1)). In an effort to understand the proximal development of STBs, several influential theoretical models have been proposed. Prominent suicide theories conceptualize STBs as emerging through relatively linear processes driven by specific risk factors, such as hopelessness, thwarted belongingness, or feelings of entrapment (2–4). Despite their substantial contributions to the field, the prediction of suicidal behavior is still challenging (5). Moreover, both existing theories and the empirical evidence provide comparatively limited insights into the underlying proximal psychological processes that characterize the emergence and subjective experience of a suicidal crisis.

Among the psychological factors implicated in suicidal crises, affective states appear to play a central role. A growing body of evidence indicates that elevated negative affective states have been consistently associated with increases in suicidal thoughts (6, 7). In a study by May and colleagues (8), 95% of individuals experiencing suicidal thoughts reported a desire to escape from negative affect as a main reason for contemplating suicide. Together, these findings suggest that the ability to regulate intense negative emotions may be central to understanding the emergence and maintenance of suicidal thoughts. Emotion regulation broadly refers to the processes by which individuals influence the experience, intensity, duration, and expression of their emotions. It has been conceptualized as a goal-directed process involving the identification, selection, and implementation of strategies aimed at modifying emotional experiences (9).

Regarding STBs, evidence indicates that emotion regulation difficulties, including limited emotional awareness, are associated with the frequency of suicidal thoughts and attempts (10, 11). Moreover, meta-analytic evidence showed that ERS that are typically considered as maladaptive (e.g., rumination, expressive suppression) are positively associated with STBs, whereas strategies that are considered as adaptive (e.g., cognitive reappraisal, mindfulness) are negatively associated with STBs (12). However, most studies on emotion regulation and STBs have relied on self-report questionnaires (10, 12, 13), thereby providing only limited insights into how emotion regulation unfolds in the onset and during suicidal crises and which strategies individuals experiencing STBs actually employ.

To date, only two studies have examined emotion regulation in individuals with STBs, using ecological momentary assessments (EMA). The first study (14) investigated frequently recommended strategies in an adult sample with Borderline Personality Disorder (BPD). Results indicate that self-care (“Did something good for myself.”) was the only strategy that reduced negative affect, controlling for all other strategies. This finding provides valuable insights into the effectiveness of commonly recommended strategies by showing that most of the highly recommended strategies (e.g., distraction, acceptance) were not effective in reducing negative affect in the context of BPD. The second EMA study examined a set of ERS that are typically considered as adaptive (e.g., cognitive reappraisal) versus maladaptive (e.g. rumination) in individuals with and without STBs (15). Compared with individuals with psychiatric disorders but no history of STBs, participants with STBs reported higher regulatory effort and substance use. Compared with healthy controls, they reported greater distraction and rumination, as well as lower self-regulatory success. Additionally, self-injurious behavior and substance use were uniquely associated with suicidal thinking.

While these studies represent an important step toward capturing emotion regulation in daily life in individuals with STBs, quantitative EMA research typically relies on predefined sets of commonly used and well-established strategies, thereby prioritizing broadly applicable behaviors while providing limited insight into individual, idiosyncratic patterns of regulation. Moreover, EMA designs are inherently constrained in the number and type of strategies that can be assessed in order to minimize participant burden. Accordingly, they may overlook strategies that are personally meaningful, context-specific, or less commonly studied, particularly in high-risk populations such as individuals experiencing STBs. In this regard, qualitative studies have explored individuals with lived experience of STBs (16–18), including how individuals describe their emotional distress and their perceived needs during suicidal crises. However, these studies tended to focus on the broader subjective experience of suicidal crises, rather than systematically examining the range of ERS (17). Thus, an in-depth exploration of ERS and their perceived effectiveness during suicidal crises remains limited.

While emotion regulation primarily concerns the modulation of affective states, coping with suicidal thoughts requires responding to suicidal thoughts and urges themselves. Accordingly, beyond the regulation of negative affective states, individuals experiencing STBs must also contend with the presence of suicidal thoughts and urges. How they respond to these experiences, and which strategies they employ in dealing with them, remains insufficiently understood. In this context, suicide-specific coping refers to the cognitive, behavioral, and interpersonal strategies individuals use to manage, reduce, or tolerate suicidal thoughts and urges (19). This is particularly relevant given that suicidal thoughts constitutes a risk factor for suicide attempts and suicide death (20, 21). Understanding how individuals cope with suicidal thoughts may therefore be critical for identifying processes that either intensify or attenuate risk and may thus inform suicide specific psychotherapeutic interventions. However, there is little empirical evidence addressing this question. Examining suicide-specific coping may help explaining why some individuals do not progress to suicide attempts despite experiencing high levels of suicidal thoughts, whereas others with comparable levels of thoughts do.

For example, Simon and colleagues (22) used self-report measures to assess how useful individuals with life-time STBs rated certain coping strategies for dealing with suicidal thoughts. Results indicated that common suicide prevention strategies (e.g., distraction, family support) were found to be of limited help. Furthermore, a recent study by Interian and colleagues (23) assessed individuals’ ability to cope with suicidal thoughts and urges using self-report measures and found that greater adaptive coping was associated with a significantly reduced likelihood of a post-discharge suicide attempt among veterans. Beyond studies using self-report questionnaires, empirical evidence on suicide-specific coping strategies (SCS) in daily life is scarce, with only two EMA studies addressing this topic. The first study found that predefined ERS (e.g., “keeping busy,” “positive thinking,” “finding perspective”) were associated with reduced suicidal thoughts in adults with BPD (24). The second study showed that greater use of adaptive coping strategies (e.g., personal support, cognitive strategies) was associated with lower suicidal urges the next day in adolescents with STBs (25).

As with ERS, existing research on SCS lacks a comprehensive understanding of the range of strategies individuals employ, how they are chosen in specific situations, and how they unfold and are experienced during a suicidal crisis. Qualitative approaches are particularly well suited to address these gaps, as they can provide a nuanced understanding of strategy use, contextual factors, and perceived effectiveness, as well as insight into the dynamic self-regulatory processes underlying the management of emotional distress and suicidal thoughts during suicidal crises. Such insights are essential for refining theoretical models of self-regulatory processes in the context of STBs, guiding future studies on the topic, and for developing effective interventions.

The study is conceptually informed by the Dual-System Model of Suicidality [DSMS (19)]. The model conceptualizes suicidal crises as emerging from sustained negative affect in interaction with deficits in self-regulatory processes, including difficulties in emotion regulation and suicide-specific coping. Importantly, the DSMS highlights that situational and contextual factors can influence how individuals respond to emotional distress and suicidal urges, including both the availability of regulatory resources and the selection of coping strategies. Within this framework, self-control represents a key self-regulatory capacity that may vary across situations. Self-control is defined as the capacity to override impulses, suppress urges, and resist temptations and contributes to adaptive goal pursuit (26). Conceptually, a distinction can be made between trait self-control, referring to relatively stable individual differences, and state self-control, reflecting momentary fluctuations in available regulatory resources (27). Reduced state self-control can be conceptualized as a situational constraint that limits adaptive coping and promotes reliance on more immediate, less adaptive regulation strategies (28). As resources become depleted, individuals increasingly rely on maladaptive strategies and exhibit reduced impulse inhibition (29, 30), which is particularly relevant for suicidal impulses.

Against this background, the present qualitative study aimed to explore (1) which strategies individuals with STBs use to regulate *negative affective states* and how they evaluate their effectiveness, (2) which strategies they employ in coping with *suicidal thoughts and suicidal urges*, and how they perceive their impact, and (3) which *situational and contextual factors* they consider relevant when choosing strategies to regulate negative affective states and to cope with suicidal thoughts and urges, with a particular emphasis on *perceived self-control capacity*.

## 2 Methods

### 2.1 Participants

Participants were recruited between March 2023 and January 2025 from inpatient psychiatric units in Leipzig and Bernburg, Germany. Eligible participants were adults admitted due to an acute suicidal crisis or following a recent suicide attempt prior to hospital admission. Additional inclusion criteria were a minimum age of 18 years and sufficient German language skills. Individuals with current psychotic symptoms, significant cognitive impairment, or reduced responsiveness due to severe physical injuries were excluded from participation. The sample consisted of 12 inpatients (58.3% women, n = 7; 41.7% men, n = 5), aged 21 to 61 years (M = 36.3, SD = 13.4). Among the 12 participants, 4 (33.3%) had attempted suicide prior to admission, and 8 (66.7%) had a history of suicide attempts. Furthermore, 9 participants (75%) were not in a romantic relationship, and the same proportion had no children. Six participants (50%) were living alone, while the remaining lived with others. Regarding employment status, 6 (50%) were unemployed, 3 (25%) were employed, and 1 (8.3%) each was retired, a student, or on sick leave. With respect to clinical diagnoses, 6 (50%) participants were diagnosed with depression, 5 (41.7%) with personality disorders, and 1 (8.3%) with post-traumatic stress disorder. Regarding mental health treatment within the three months preceding admission, two participants (16.7%) had received inpatient treatment, two (16.7%) were receiving outpatient psychiatric care, one (8.3%) was engaged in outpatient psychotherapy, and one (8.3%) was receiving support from a community psychiatric service.

### 2.2 Procedure

Potential participants were screened for eligibility according to the predefined inclusion criteria. All individuals who met these criteria and gave written informed consent were enrolled in the study. Two individuals were excluded because they did not report current suicidal thoughts at the time of assessment. All participants received information on the content and procedure of the interview, the aim of the study, data protection policies, the voluntary nature of participation prior to giving informed consent. Upon completion of the questionnaires assessing socio-demographic and clinical characteristics, participants were scheduled for a qualitative interview. Clinical diagnoses were obtained from medical records. The semi-structured interviews were conducted on the psychiatric wards by the first (JB) and the second author (JP). The interviews were audio-recorded and lasted approximately 90 minutes. Following the interview, participants were asked about their current emotional state and whether they were experiencing emotional distress or suicidal intent. None of the participants reported acute emotional distress or suicidal intent following the interview. All participants received €30 compensation for their study participation. The study received ethical approval from the ethics committee of the Medical Faculty of University of Leipzig (no. 335/22-ek).

### 2.3 Measures

For the interviews, a semi-structured interview guide was used (see **Supplementary Material**), beginning with an open-ended prompt: “Please describe, in your own words, how your suicidal crisis or suicide attempt developed.” Following the initial open-ended prompt and participants’ responses, the interview continued with a more structured section informed by the DSMS, focusing on negative affective states, emotion regulation strategies, and participants’ strategies to cope with suicidal thoughts and urges. Furthermore, participants were asked about the factors they perceived as influencing their selection of ERS and SCS.

### 2.4 Data analysis

The interviews were audio-recorded and transcribed with the transcription software f4x (31). Data analysis was conducted using the content structuring qualitative content analysis according to Kuckartz and Rädiker (32). MAXQDA version 26.0 was used for computer-assisted coding (33).

The category system was developed using a combined deductive-inductive approach. Deductive coding domains were derived from the interview guide, research questions, and relevant theoretical and empirical literature. These broad domains included emotion regulation strategies, suicide-specific coping strategies, contextual influences on strategy use, and the role of self-control. To support a consistent coding process, coding rules were defined regarding the assignment of text segments, category boundaries, and coding consistency across interviews. Following an initial familiarization with the material, brief case summaries were prepared for each interview. The second author (JP) then coded the interviews using the deductively derived coding domains. Relevant text passages were extracted, paraphrased, and summarized. Based on these data, recurring themes and strategy patterns were identified and organized into inductively developed subcategories. Throughout the analytic process, categories, subcategories, and coding rules were iteratively refined, differentiated, and expanded to best represent participants’ accounts while maintaining consistency across the dataset. While the broad coding domains were informed by the interview guide and the DSMS, the specific strategies, subcategories, and thematic patterns reported in the Results emerged inductively from participants’ narratives and were iteratively refined throughout the analysis. This approach allowed the theoretical framework to guide the initial organization of the analysis while ensuring that the findings remained grounded in participants’ accounts.

To enhance the reliability and objectivity of the coding process, two interviews (corresponding to 16.7% of the total data set) were independently coded by the first and the second author (JB and JP) at the beginning of the coding process. Coding discrepancies were subsequently discussed and resolved by consensus, and category definitions were refined where necessary. The development of the category system was further discussed in peer debriefings, and feedback was incorporated during the iterative refinement process. The iterative refinement of categories and subcategories continued until thematic saturation was reached. Saturation was determined when no new subcategories emerged from the interview material and additional interviews only contributed further examples or elaborations of existing categories rather than novel thematic content. In a final step, the entire data set was analyzed using the finalized category system. All identified categories were included in the results section, including those with low coding frequency, to reflect the exploratory aim of the study and to capture the full range of reported strategies. For the purpose of this article, the quotations were translated into English.

### 2.5 Researcher characteristics

The first author (JB) conducted six interviews and independently coded two interviews. She is a 41-year-old Western European licensed cognitive-behavioral psychotherapist and researcher with 15 years of clinical and research experience in the field of suicidality. The second author (JP) conducted eight interviews, coded and analyzed the data. She is a 28-year-old Western European master’s student in Clinical Psychology and Psychotherapy with clinical experience working with individuals with STBs as an intern and research assistant in suicide research projects. Several measures were implemented to enhance methodological rigor and minimize the influence of subjective assumptions on data interpretation. These included the combined deductive–inductive development of the category system, independent coding of a subset of interviews, consensus discussions of coding discrepancies, and peer debriefings with qualitative researchers from the department. Throughout the analytic process, alternative interpretations of the findings were critically discussed and incorporated into the ongoing refinement of the coding framework.

## 3 Results

### 3.1 Emotion-regulation strategies

Across all interviews, participants reported experiencing intense negative affect prior to the suicidal crisis, which intensified over time and culminated in a suicidal crisis. Initially, participants described emotions such as anxiety, sadness, self-doubt, disappointment, and guilt. As the crisis progressed, feelings of overwhelm, despair, shame, and anger were reported, in some cases escalating to self-hate.

For systematic classification, the reported ERS were categorized according to their functional orientation into approach-oriented and avoidance-oriented strategies. The categorization of ERS into approach-oriented and avoidance-oriented strategies draws on established approach–avoidance frameworks in coping research (34). *Approach-oriented ERS* involve engaging with and processing distressing thoughts or emotions, whereas *avoidance-oriented ERS* aim to reduce or escape from internal experiences without directly addressing them. A distinction between adaptive and maladaptive strategies was deliberately avoided, as participants frequently described the effectiveness of strategies as context-dependent and at times ambivalent. Therefore, the classification focused on functional orientation rather than on *a priori* assumptions about adaptiveness.

In total, 15 distinct emotion regulation strategies were identified (see **Table 1**). All twelve participants reported using both approach-oriented (at least one) and avoidance-oriented strategies (at least five different strategies). Avoidance-oriented ERS (n = 142 coded segments) were more frequently reported than approach-oriented ERS (n = 62 coded segments).

**Table 1:** Coding frequencies of emotion-regulation strategies.

Emotion-regulation strategies	Number of coded segments	Int 01	Int 02	Int 03	Int 04	Int 05	Int 06	Int 07	Int 08	Int 09	Int 10	Int 11	Int 12	Number of participants
*Avoidance-orientated*	**142**	**14**	**16**	**6**	**11**	**15**	**7**	**8**	**12**	**7**	**16**	**13**	**17**	**12**
Suicidal thoughts	31	3	4	2	3	1	0	2	3	2	3	4	4	11
Suppression	24	3	1	1	2	3	2	1	3	0	1	2	5	11
Self-devaluation	20	0	2	1	1	4	1	2	1	0	4	2	2	10
Rumination	17	1	0	1	2	6	2	0	1	1	2	1	0	9
Withdrawal	13	1	3	0	1	1	1	1	1	0	1	2	1	10
Substance use	11	3	2	0	1	0	1	0	0	0	2	0	2	6
Self-injury	9	0	1	0	1	0	0	1	0	1	0	2	3	6
Distraction	9	2	1	1	0	0	0	1	1	2	1	0	0	7
Escape	8	1	2	0	0	0	0	0	2	1	2	0	0	5
*Approach-oriented*	**62**	**8**	**7**	**5**	**1**	**4**	**4**	**3**	**12**	**3**	**3**	**6**	**6**	**12**
Self-disclosure	18	0	4	1	0	1	1	1	5	2	1	2	0	9
Seeking support	13	2	1	1	1	1	0	0	3	0	0	1	3	8
Confrontation	10	3	0	2	0	0	2	0	1	1	1	0	0	6
Emotional expression	9	0	1	1	0	1	0	1	1	0	0	3	1	7
Cognitive reappraisal	7	3	1	0	0	0	0	0	1	0	0	0	2	4
Activation	5	0	0	0	0	1	1	1	1	0	1	0	0	5

Int, Interview.

Bold values represent totals for each higher-order category. Bold values in the interview columns (Int 01–Int 12) and the “Number of coded segments” column indicate the number of coded segments per interview and across all interviews, respectively. In the “Number of participants” column, the bold value indicates the number of participants who reported at least one strategy within the respective higher-order category.

#### 3.1.1 Approach-oriented emotion-regulation strategies

Approach-oriented ERS comprised six distinct strategies (see **Table 1**), with self-disclosure and seeking support being the most frequently reported.

##### 3.1.1.1 Self-disclosure

Self-disclosure emerged as the most prominent approach-oriented strategy, with nine participants describing instances in which they shared their emotional states with others. This strategy involved communicating one’s emotional distress to others with the primary aim of receiving understanding, validation, or relief. Self-disclosure was predominantly directed toward close others (e.g., family members, friends, partners) who were easily accessible to the participants. Two temporal patterns were frequently described: self-disclosure was employed both at the onset of increasing emotional distress and at critical transition points toward acute emotional crises. It often happened during a crisis when participants were not yet able to clearly understand their emotional states and therefore relied on external feedback or resonance, as one participant stated:

“Nothing was working anymore. I felt like I was doing everything wrong. So I went to my boss’s office and, for the first time, broke my silence and said, ‘I don’t know what’s going on with me.’ At that point, I didn’t really know anything about this illness or what was happening to me.” (int 11)

Overall, the self-disclosure was perceived as helpful when it was met with a responsive and validating reaction. However, particularly in the early stages of distress, several participants reported experiencing minimizing or dismissive responses, which, from their perspective, inhibited further self-disclosure.

##### 3.1.1.2 Seeking support

The strategy of seeking support was reported by eight participants and involved reaching out to individuals within their private or professional networks (e.g., general practitioners, psychotherapists) to cope with emotional distress. In contrast to self-disclosure, which primarily aimed at emotional validation, seeking social support was directed toward obtaining specific assistance, advice, or guidance.

Participants emphasized that support from close others could serve as an immediately accessible resource during periods of emotional distress. For example, one participant described contacting friends to avoid being alone with distressing feelings:

“I try to reach out to friends when I feel very sad, so that I’m not alone.” (int 04)

Participants further described that seeking support was particularly helpful when professional or personal social support was easily accessible and required little effort. The strategy was experienced as alleviating distress and providing a sense of not having to cope with difficulties alone. However, when access to professional help involved substantial barriers, such as prolonged waiting times, the attempt to seek support itself could become a source of distress:

“I’ve been looking for a psychotherapist who can offer me weekly appointments for a year now. Now I’ve been told to wait until July again, and it’s only March. And nothing is moving forward. And I’m so afraid of going back to work [ … ]. Then I’ll just slip right back into it again.” (int 03)

Rather than providing relief, these structural barriers intensified feelings of frustration, helplessness, and hopelessness.

In addition to the core strategies described above, participants reported several other approach-oriented ERS, particularly emotional expression, cognitive reappraisal, activation, and confrontation. Emotional expression referred to deliberately releasing emotions (e.g., crying or shouting) to reduce emotional tension. While this was sometimes experienced as relieving, it was also described as occurring during impulsive affective outbursts associated with a perceived reduction in self-control and escalating suicidal urges. Cognitive reappraisal involved reinterpreting situations or adopting alternative perspectives in order to better cope with distressing emotions. For example, one participant described deliberately shifting perspective by asking themselves:

“(…) I then go through it in my head and ask myself, ‘Okay, what would my everyday hero do right now?’” (int 12)

It was typically used after an initial calming of affect, when cognitive resources became available, and was generally perceived as effective. Activation referred to engaging in goal-directed activities, such as physical exercise or creative tasks, to interrupt rumination and to reduce tension. Although activation may partly involve elements of distraction, participants primarily described it as an active attempt to regulate negative affective states and to regain behavioral control. Participants reported that this strategy was most helpful when distress had not yet reached its peak and they still felt capable of taking action. Confrontation involved deliberately engaging with and tolerating negative affective states (e.g., intense anxiety) rather than avoiding them. This strategy was often experienced as overwhelming and less effective when individuals remained in states of intense distress without external support or coping plans.

#### 3.1.2 Avoidance-oriented emotion-regulation strategies

Participants predominantly reported strategies aimed at short-term tension reduction or distancing themselves from distressing emotions without addressing their underlying causes. In total, nine avoidance-oriented ERS were identified (see **Table 1**). The core strategies included suicidal thoughts, suppression, and self-devaluation.

##### 3.1.2.1 Suicidal thoughts

The most frequently reported ERS involved the use of suicidal thoughts, which participants described as a way of coping with negative affect. Eleven of the twelve participants described suicidal thoughts as a form of “relief” or “escape fantasy.” One participant, however, described their suicide attempt as an impulsive act that occurred without preceding suicidal thoughts. For the remaining participants, the perceived cognitive availability of suicide as an option was described as temporarily reducing acute negative affect and providing a subjective sense of control, as illustrated by two participants:

“Often, they were simply feelings that I couldn’t control in that moment and that felt overwhelming, and I just wanted to escape from them. Especially when I was feeling self-hatred or sadness, suicidal thoughts felt like a mental escape route. It was as if everything seemed hopeless, but at the same time I felt that I still had an emergency exit.” (int 04)

“So, it wasn’t always connected to actions; often it was just feelings that I couldn’t control in that moment and that felt overwhelming, and I simply wanted to escape from them. But you can’t escape from your feelings. And they always led me into despair, which then brought me to suicidal thoughts.” (int 02)

Similarly, another participant described the perceived availability of suicide as a source of relief:

“For me, it’s a way of coping (…) knowing that I still have the option to do it if things become too overwhelming gives me a sense of relief.” (int 12)

Although participants described suicidal thoughts as temporarily relieving and emotionally calming, over time they perceived an intensification of these thoughts and an increased susceptibility to suicidal impulses, highlighting the potentially harmful effects of this strategy in the longer term (negative reinforcement). Participants further reported that suicidal thoughts were particularly likely to emerge during periods of heightened vulnerability, such as following critical life events, increasing symptom burden, or when effective ERS were lacking.

##### 3.1.2.2 Suppression

The suppression or concealment of negative emotions, aimed at both hiding them from others and denying them to oneself, was reported by eleven of the twelve participants. Emotional suppression often occurred following critical life events and was facilitated by perceived external expectations or pressure. In the short term, participants described this strategy as enabling them to maintain outward functioning despite internal distress:

“Well, mainly from the outside it always looked like that. At least a friend once told me that I actually seemed happy, but inside I always felt terrible.” (int 02)

However, repeated reliance on this strategy was experienced as increasingly resource-depleting. Over time, participants reported that suppressing emotions no longer reduced distress but instead contributed to a gradual intensification of negative affect.

##### 3.1.2.3 Self-devaluation

Ten participants described engaging in negative self-attributions. This strategy was primarily employed during periods of increasing distress, declining functioning, and when previously used ERS were perceived as ineffective. Participants described using self-devaluing thoughts to cope with or compensate for feelings of shame and guilt, and to avoid confronting internal conflicts or interpersonal tensions. One participant stated:

“Out of sheer exhaustion, I kept asking myself what my purpose even was anymore, why I was still here, and whether I meant anything to anyone at all.” (int 08)

Participants reported that this pattern of negative self-evaluation often intensified negative affect, sometimes escalating into self-hate. At the same time, it was experienced as limiting the use of approach-oriented ERS such as self-disclosure, while tendencies toward withdrawal and suicidal cognitions often increased.

In addition to the core avoidance-oriented strategies described above, participants reported further strategies aimed at distancing themselves from distressing emotional experiences. These included rumination, social withdrawal, substance use, non-suicidal self-injury, distraction, and escape (see **Table 1**). Rumination was described as repetitive and circular thinking about causes and consequences of distress and was often accompanied by self-blame and catastrophic thoughts. Although it was sometimes experienced as an attempt to gain control over negative emotions, participants reported that it typically intensified emotional distress and contributed to sleep and concentration difficulties. One participant said:

“I just couldn’t cope with it anymore. All I wanted was to drive a knife into my head so that the thoughts would stop. The pressure inside my head was unbelievable because of the constant rumination and all those thoughts. I simply couldn’t go on anymore.” (int 05)

Social withdrawal involved reducing social contacts and everyday activities in order to avoid overwhelming or evaluative situations, but was frequently associated with increased isolation, loss of daily structure, and intensified rumination. Substance use, including alcohol, drugs, or medication, was used by some participants to temporarily reduce intense emotions or intrusive thoughts, but often resulted in loss of control, interpersonal conflicts, and increased suicidal thoughts. Similarly, non-suicidal self-injury, distraction (e.g., media use or engagement in work or household tasks), and escape, understood as leaving or distancing oneself from situations perceived as overwhelming or uncontrollable, were described as strategies aimed at short-term tension reduction or emotional distancing. While these strategies were sometimes experienced as immediately relieving, participants frequently described them as contributing to a cycle of increasing emotional burden and reduced access to more adaptive coping strategies.

**Figure 1:**
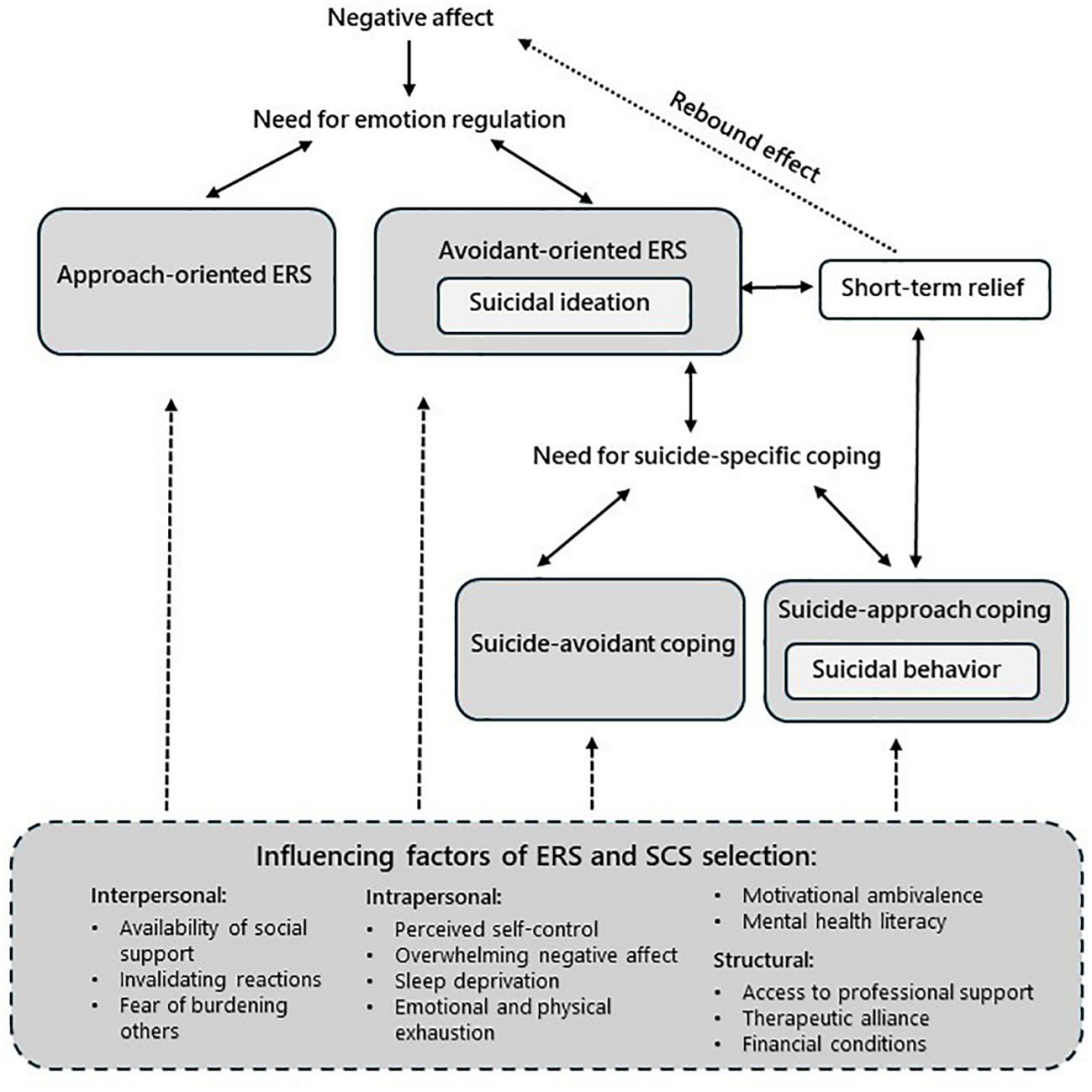
Participant-derived conceptual model of emotion regulation and suicide-specific coping during suicidal crises. This figure presents a participant-derived conceptual model summarizing themes identified across interviews and their interpretation within the framework of the Dual-System Model of Suicidality (DSMS). The model integrates participants’ descriptions of negative affect, emotion regulation strategies (ERS), suicide-specific coping strategies (SCS), and contextual influences on coping during suicidal crises. ERS and SCS represent analytical categories developed by the authors to organize the reported strategies. The concepts of an increasing need for emotion regulation, a need for suicide-specific coping, and the proposed sequence of processes represent theoretical interpretations informed by the DSMS and the qualitative findings. The arrows represent the authors’ theoretically informed integration of the identified themes and should not be interpreted as empirically validated causal or temporal relationships. Future prospective studies are needed to examine the temporal dynamics of these processes.

Overall, participants described a predominant reliance on avoidance-oriented ERS prior to suicidal crises. Barriers to the use of approach-oriented strategies, such as lack of support and invalidating reactions often led participants to rely increasingly on avoidance-oriented regulation. Although these strategies were frequently described as providing rapid short-term relief, participants also reported that they were often followed by increasing distress and a reduced ability to cope with emotional challenges. Taken together, these accounts suggest a pattern in which avoidance-oriented regulation may provide temporary relief while being perceived as contributing to the persistence or intensification of distress over time (see **Figure 1**).

### 3.2 Suicide-specific coping

Beyond strategies aimed at regulating negative emotions, participants also described specific coping strategies used to manage suicidal thoughts and urges. Notably, several strategies (e.g., withdrawal, suppression, and substance use) were described in both emotion regulation and suicide-specific coping contexts. While their behavioral expression was similar, their function differed depending on whether they were used to regulate negative affect or to manage suicidal thoughts and urges.

In total, 17 suicide-specific coping strategies (SCS) were identified across 258 coded segments (see **Table 2**). These strategies were categorized into suicide-avoidant and suicide-approach coping strategies. This distinction differs conceptually from the classification of ERS, as it refers to different points of reference. While the ERS classification is based on the direction of regulation in relation to emotional experiences (approach vs. avoidance of emotions), the categorization of SCS reflects the motivational orientation toward suicidal behavior.

**Table 2:** Coding frequencies of suicide-specific coping strategies.

Suicide-specific coping strategies	Number of coded segments	Int 01	Int 02	Int 03	Int 04	Int 05	Int 06	Int 07	Int 08	Int 09	Int 10	Int 11	Int 12	Number of participants
*Approach-oriented*	**72**	**3**	**6**	**7**	**8**	**3**	**0**	**2**	**1**	**8**	**16**	**5**	**13**	**12**
Justifying suicide	26	0	3	5	2	2	0	0	0	1	6	3	4	8
Suicide-related preparatory behavior	15	2	0	1	3	1	0	0	0	2	4	1	1	8
Withdrawal	8	0	2	0	2	0	0	0	0	0	3	0	1	4
Distraction	6	0	0	0	0	0	0	0	1	1	0	1	3	4
Suppression	8	1	0	0	1	0	0	1	0	2	1	0	2	6
Substance use	4	0	1	0	0	0	0	1	0	0	1	0	1	4
Suicidal behavior	5	0	0	1	0	0	0	0	0	2	1	0	1	4
*Avoidance-oriented*	**186**	**11**	**9**	**18**	**6**	**17**	**13**	**9**	**8**	**20**	**25**	**20**	**30**	**12**
Professional support	42	2	0	2	2	5	2	4	1	6	7	4	7	11
Social support	27	1	3	4	1	4	2	2	1	2	2	4	1	12
Disclosing suicidal thoughts	20	1	3	3	1	0	1	0	0	3	2	2	4	9
Focusing on reasons for living	19	2	1	1	0	2	1	0	4	0	2	5	1	9
Questioning the desire to die	15	1	0	2	0	0	0	0	0	5	0	0	7	4
Self-soothing	14	0	0	2	1	2	0	1	0	0	3	4	1	7
Problem identification	14	0	2	1	1	2	4	1	0	0	2	0	1	8
Future planning	14	0	0	0	0	1	2	0	2	4	4	0	1	6
Other safety strategies	11	2	0	2	0	0	0	0	0	0	3	0	4	4
Emergency contacts	10	2	0	1	0	1	1	1	0	0	0	1	3	7

Int, Interview.

Bold values represent totals for each higher-order category. Bold values in the interview columns (Int 01–Int 12) and the “Number of coded segments” column indicate the number of coded segments per interview and across all interviews, respectively. In the “Number of participants” column, the bold value indicates the number of participants who reported at least one strategy within the respective higher-order category.

*Suicide-avoidant strategies* refer to coping efforts aimed at creating motivational distance from suicidal behavior, whereas *suicide-approach strategies* involve forms of coping that orient individuals toward suicide or maintain its perceived availability as an option. Notably, some strategies that may appear avoidant at the first glance (e.g., distraction) were classified as *suicide-approach strategies* because they did not involve an active disengagement from suicidal intent and were described as ultimately facilitating a continued orientation toward suicidal behavior.

Overall, suicide-avoidant strategies (n = 186 coded segments) were reported considerably more often than suicide-approach strategies (n = 72 coded segments) as presented in **Table 2**.

#### 3.2.1 Suicide-approach coping strategies

##### 3.2.1.1 Justifying suicide

Eight participants described the absence of a future perspective, while repeatedly reflecting on the perceived meaninglessness of their lives. These reflections often involved thoughts that death would represent relief from ongoing distress. Importantly, participants described these cognitions not only as expressions of emotional suffering but also as a way of cognitively engaging with and evaluating their desire to die. In this sense, these reflections served as a SCS by helping participants make sense of their suicidal intent and justify their wish to die to themselves, as one participant described:

“I keep falling back into this hole again: ‘What if I just wasn’t here anymore? Then I wouldn’t have to go through this therapy. Then I’d be free. Then I could finally decide for myself and be the way I want to be, instead of letting others decide for me.’”(int 03)

Such thoughts were frequently associated with cognitive narrowing toward suicidal behavior and an increased desire to die. Several participants described these reflections as preceding suicide-related preparatory behaviors.

##### 3.2.1.2 Suicide-related preparatory behavior

Suicide-related preparatory behaviors were reported by eight participants as a coping strategy with regulatory function. These behaviors included searching for information about lethal suicide methods, obtaining or preparing means, writing farewell letters, or arranging matters related to death. Participants described these behaviors as typically occurring after prolonged rumination, social withdrawal, and increasing suicidal thoughts. Engaging in preparatory behavior was often associated with a perceived sense of control over a subjectively uncontrollable and exhausting situation, yet simultaneously increased the urge to engage in suicidal behavior. From the participants’ perspective, these preparations often represented a critical threshold characterized by immediate proximity to suicidal behavior. As one participant described:

“It starts with the thoughts. Then comes the preparation phase. So, how do I do it? [ … ] So, as I said, that’s the escalation from the thoughts: I feel worthless because of anger, fear, and sadness, and it tears me apart. But the escalation of that is making plans. That means, okay, I want to do it now, and I can come up with a plan very quickly. And that includes, for example, writing a goodbye letter.” (int 03)

In addition to the most frequently reported strategies, several further suicide-approach coping strategies were identified, including withdrawal in response to suicidal thoughts, suppression, distraction, substance use, and suicidal behavior.

*Withdrawal* in response to suicidal thoughts referred to the deliberate avoidance of social contacts and everyday activities in order to conceal suicidal thoughts and prevent confrontation or interventions by others. In contrast to withdrawal as an ERS, which primarily served to cope with general emotional overload, this form of withdrawal was specifically aimed at hiding suicidal thoughts and intent, and was often accompanied by planning or preparatory behaviors.

*Suppression* involved the intentionally suppressing of suicidal thoughts and urges or denying their severity in order to maintain the appearance of functioning or to avoid interventions such as hospitalization. Participants described this strategy as cognitively demanding and reported that prolonged use was often accompanied by a perceived decline in their ability to control suicidal thoughts and urges.

*Distraction* referred to attempts to temporarily distance oneself from suicidal thoughts through activities such as excessive media use or sleeping. While this strategy was sometimes experienced as temporarily relieving, participants reported that it did not address the underlying drivers of suicidal thoughts. Therefore, distraction was categorized as a suicide-approach coping strategy, as it did not appear to alter the underlying motivational state or address the processes contributing to suicidal thoughts.

*Substance use* was described by some participants as a rapid means of dampening acute suicidal thoughts. However, participants frequently reported that, once its effects subsided, this strategy was accompanied by a diminished perceived ability to control suicidal thoughts and urges, often resulting in a subsequent intensification of suicidality.

*Suicidal behavior*, was described by four participants as an attempt to escape from a negative affective state. Although participants who used this behavior as a coping strategy reported short-term relief, these behaviors were often followed by intense feelings of guilt, shame, and self-devaluation, suggesting a paradoxical increase in distress over time.

Across participants, a recurring sequence of suicide-approach coping strategies was described. Withdrawal and suppression often marked the beginning of this process, followed by distraction as a temporary coping attempt. In situations of persistent distress, substance use sometimes became part of this pattern. This constellation was associated with increasing negative affect and suicidal thoughts, which participants perceived as facilitating the transition toward justifying suicide, suicide-related preparatory behaviors, and, in some cases, suicidal behavior.

#### 3.2.2 Suicide-avoidant coping strategies

In contrast to suicide-approach coping strategies, participants also described several suicide-avoidant coping strategies aimed at increasing motivational distance from suicidal behavior. Some strategies were described as preventive attempts to maintain safety during escalating crises, whereas others were reported *as responses following* suicidal behavior or during recovery phases. In total, ten strategies were identified, with social and professional support and suicide-specific self-disclosure being the most frequently reported (see **Table 2**).

##### 3.2.2.1 Disclosing suicidal thoughts

Nine participants reported disclosing suicidal thoughts or urges to trusted individuals, such as close others or professional contacts, and some also described exchanges with other affected individuals as helpful. In contrast to self-disclosure as an ERS, which primarily involved communicating emotional distress, this strategy explicitly focused on revealing suicidal thoughts or intentions. The primary aim was to obtain attention for their situation and emotional relief, for example through feeling seen, reassured, or connected to others. Participants described using this strategy particularly during phases of reduced perceived self-control while still weighing life and death, often accompanied by shame and concerns about burdening others. As one participant stated:

“I try to talk to my partner, which is very, very difficult for me, because I keep telling myself that I’m burdening him with it.” (int 10)

The perceived benefit of this strategy depended on the availability and the reactions of others. When responses were perceived as invalidating, participants reported being less willing to disclose suicidal thoughts again.

##### 3.2.2.2 Professional and social support

Almost all participants reported seeking formal or informal support to cope with suicidal thoughts and urges. Formal support included professional services such as psychotherapeutic or psychiatric treatment as well as consultations with general practitioners. Participants described seeking such help either on their own or with the support of others, often following suicide-related preparatory behaviors or after a suicide attempt. Professional support was generally perceived as helpful through psychotherapeutic or pharmacological treatment, and helping to establish safety plans, and a greater structure and routine in daily life. However, participants also reported critical barriers such as long waiting times, feelings of shame, concerns about stigma, or fear of coercive treatment.

In addition to formal services, participants described receiving support from their social environment, including practical help, accompaniment to appointments, and assistance with structuring daily routines. Such support was often reported after prior suicide-specific self-disclosure and was experienced as stabilizing and relieving.

##### 3.2.2.3 Focusing on reasons for living and questioning the desire to die

Participants described cognitive strategies that involved actively reflecting on motives related to suicidal behavior. These included intentionally focusing on reasons for living, such as personal values, life meaning, or significant others, as well as questioning whether suicide was justified or truly the only remaining option in the given situation. Participants often referred to an internalized sense of responsibility toward their social environment, sometimes experienced as an inner voice of significant others reminding them of the potential consequences of their actions. As one participant described:

“The inner voice then says: ‘Your father wouldn’t have wanted this. You would make your family sad. Your last friends who are still there.’” (int 01)

Such reflections were frequently reported during phases of acute suicidality and in some cases contributed to delaying or aborting a suicide attempt. Participants also described attempts to question their suicidal intent by reflecting on the expected consequences of suicidal actions or considering whether the situation was truly unbearable enough to justify suicide. Three of the four participants who reported using this strategy described that pausing to reflect reduced the urge to engage in suicidal behavior. However, when participants were already cognitively focused on carrying out a suicidal act, these reflections sometimes became part of a recurring cognitive loop that ultimately reactivated suicidal planning and the motivation to act on suicidal urges. As one participant explained shortly before a suicide attempt:

“Then I sat there for a while and really thought about it, asking myself whether it was actually such a bad idea or not. Then the thoughts came back, it all started again, the plans returned, the knife was already there, and eventually I did it.” (int 03)

Although these strategies were categorized as suicide-avoidant coping, their effectiveness should be viewed as ambivalent, as they could both interrupt and, in some cases, reinforce suicidal processes (see **Figure 1**).

Participants also described several additional strategies aimed at reducing suicidal thoughts and urges. These included self-soothing strategies, such as positive self-instructions. For example, one participant described encouraging themselves to focus on manageable daily goals and maintain hope that the situation would improve:

“I told myself, ‘You have to be brave now. Set yourself something to do for the day [ … ] and little by little, things will get better.’” (int 05)

Participants further reported safety-oriented strategies, such as contacting trusted individuals or emergency services during high-risk situations, restricting access to lethal means, avoiding suicide-related online content, or taking prescribed emergency medication. These strategies were experienced as temporarily helpful in restoring a sense of self-control. However, their effects were typically short-lived, and participants reported difficulties applying them during periods of intense emotional arousal or when reasons for dying dominated.

Strategies such as problem identification and future planning were also described; however, these were mostly applied *after the acute suicidal crisis*, often with therapeutic support. In particular, problem identification was perceived as helpful, as it facilitated addressing the underlying drivers of the crisis.

Importantly, participants’ accounts suggested that the use of SCS varied across different phases of the suicidal process, highlighting the role of situational and contextual factors in shaping the selection and accessibility of coping strategies.

#### 3.2.3 Situational and contextual factors

Participants described several situational and contextual factors that influenced the selection and accessibility of strategies dealing with negative affect, suicidal thoughts and urges in suicidal crises (see **Figure 1**). These factors also shaped participants’ perceived self-control capacity, which in turn influenced the accessibility and use of ERS and SCS.

##### 3.2.3.1 Emotional and physiological distress

Participants consistently described the intensity of emotional distress as a key determinant of coping strategy selection. Increasing negative affect, experiences of overwhelm, and acute emotional dysregulation were associated with reduced access to adaptive coping strategies and a greater reliance on avoidance-oriented ERS or suicide-approach coping. Several participants reported that intense distress was accompanied by emotional numbness, making their psychological state less accessible to themselves. Physical conditions such as sleep deprivation, exhaustion, and reduced food intake were also perceived as limiting the psychological resources required to apply more effortful coping strategies such as seeking social or professional support, reflecting on reasons for living, or actively questioning suicidal intent, as one participant described:

“My body simply had very little capacity left. There were no resources available anymore. Normally, the body restores its resources through sleep, but at that point those resources were missing, which made me more vulnerable.” (int 01)

In addition, acute physiological stress responses (e.g., panic symptoms) and substance use were described as further promoting suicide-approach coping, including suicidal behavior.

##### 3.2.3.2 Cognitive factors

Participants also emphasized the role of cognitive factors in shaping coping behavior. Knowledge about mental health problems, early warning signs, and coping strategies, often acquired through psychotherapy, expanded the perceived repertoire of strategies. In contrast, limited knowledge or uncertainty about one’s own condition was associated with greater reliance on maladaptive coping. Participants further described how cognitive constriction during acute crises restricted their ability to recall or apply previously learned strategies. Low self-efficacy following repeated unsuccessful coping attempts also contributed to a shift toward suicide-approach coping strategies, as individuals increasingly perceived suicide as the only remaining option.

##### 3.2.3.3 Motivational dynamics

Motivational factors were primarily reflected in the context of suicidal ambivalence, characterized by the simultaneous presence of reasons to live and reasons to die. Participants described the relative dominance of these motives as fluctuating across situations and strongly influencing coping strategy selection. When life-oriented motives remained accessible, participants reported greater engagement in suicide-avoidant coping such as seeking support or questioning suicidal intentions. Conversely, when reasons to die became dominant, participants described withdrawing from external support and increasingly engaging in suicide-approach coping.

##### 3.2.3.4 Social and structural context

Social and structural factors also shaped coping behavior. The availability and quality of social support influenced whether participants disclosed suicidal thoughts and accessed help by others. Supportive and validating responses were described as facilitating further help-seeking and protective coping strategies, whereas invalidating reactions or concerns about burdening others often led to concealment of suicidal thoughts and increased withdrawal. Structural resources, such as access to mental health care and financial stability, were also perceived as important contextual conditions. Barriers such as long waiting times for treatment or limited financial resources were described as hindering support-seeking and prolonging crisis situations.

##### 3.2.3.5 Role of perceived self-control

Participants described perceived self-control as an important self-regulatory capacity that shaped their ability to manage suicidal thoughts and urges during suicidal crises. On the one hand, participants reported that their perceived self-control enabled them to inhibit suicidal urges and engage in safety-oriented strategies, such as contacting others or interrupting suicide-approach behaviors, as illustrated by one participant:

“I even stopped along the way at times because I just couldn’t go on. I got out of the car and left it there.” (int 10)

On the other hand, participants described deliberately exerting self-control to suppress suicidal thoughts, regulate negative affective states, or maintain functioning despite experiencing severe internal distress. Sustaining such dysfunctional and effortful control over extended periods was perceived as psychologically exhausting and, in several cases, was followed by a marked loss of control. Participants reported that prolonged suppression of distress and the concealment of suicidal thoughts depleted their coping resources, eventually leading to a rebound-like escalation of suicidal urges in which previously accessible suicide-avoidant coping strategies were no longer available. In these moments, individuals often described acting impulsively or experiencing suicidal behavior as occurring in a state of reduced or absent perceived self-control, as one participant described:

“At that point, it was just acting. Before that, when I had thought about it before, I had already held the pack of pills in my hand, but I was still able to think. I thought, ‘Oh God, my family - I can’t do this.’ But at that moment, I could only act.” (int 05)

## 4 Discussion

The present qualitative study explored how individuals with STBs regulate negative emotions, cope with suicidal thoughts and urges, how they perceive the effectiveness of these strategies, and which situational and contextual factors shape the selection of these strategies.

Four main findings emerged: First, participants described a predominance of avoidance-oriented ERS preceding suicidal crises, which were often experienced as providing short-term relief but were associated with increasing distress. Second, participants described suicidal thoughts as serving a regulatory function in response to negative affective states, providing emotional relief and a sense of control. Third, individuals described a broad range of SCS with differing motivational orientations toward suicidal behavior. Fourth, the accessibility and selection of these strategies were strongly shaped by situational and contextual factors.

### 4.1 Emotion regulation and the emergence of suicidal thoughts

A central finding of the present study is the prominent role of avoidance-oriented ERS prior to suicidal crises, which is in line with findings by Millgram and colleagues (15), who reported more frequent use of such strategies among individuals with STBs compared with healthy controls and psychiatric patients without STBs. Participants of the present study frequently described strategies such as suppression, self-devaluation, rumination, social withdrawal, or substance use as attempts to manage overwhelming emotional distress. Although these strategies often provided immediate relief or allowed individuals to maintain functioning in the short term, they were commonly perceived as contributing to an intensification of negative affect over time.

This escalating emotional burden was often described as creating conditions in which suicidal thoughts emerged as an additional strategy to cope with overwhelming distress. Almost all participants reported that thinking about suicide provided temporary relief by offering a perceived escape from distress or by restoring a sense of control over an otherwise uncontrollable situation. From a self-regulation perspective, this pattern can be understood as a self-perpetuating cycle: although suicidal thoughts provides temporary relief from distress, it works like putting a bandage on an untreated wound, thereby contributing to the persistence and escalation of emotional burden over time. This observation is consistent with findings suggesting that suicidal thoughts may function as a dysfunctional ERS in response to persistent negative affect and unsuccessful regulation efforts (8, 16). It also aligns with the DSMS (19), which conceptualizes suicidal thoughts as emerging from attempts to regulate overwhelming emotional distress while paradoxically contributing to the escalation of suicidal crises.

In contrast, participants also described a range of approach-oriented ERS, such as self-disclosure, seeking support, cognitive reappraisal, or activation. However, the use and effectiveness of these strategies appeared to depend strongly on situational and contextual conditions. For instance, self-disclosure was experienced as helpful when met with validation, but discouraging when responses were dismissive. Similarly, seeking support required the availability and accessibility of personal or professional support, and barriers to care could themselves become a source of distress. Moreover, several strategies, such as cognitive reappraisal or activation, were described as requiring a certain level of cognitive or behavioral capacity and were therefore less accessible during states of intense emotional distress or when participants perceived their self-control capacity to be reduced. Taken together, these findings suggest that although approach-oriented ERS may be effective in general, their use is constrained by internal and external factors, limiting their availability precisely at moments of heightened vulnerability.

### 4.2 Suicide-specific coping

Beyond strategies aimed at regulating negative affect, participants described a range of SCS used to manage suicidal thoughts and urges. The results of the present study showed that participants described a recurring sequence of suicide-approach coping strategies beginning with withdrawal and suppression to conceal suicidal thoughts, followed by distraction and substance use to deal with it. Distraction from suicidal thoughts and urges was perceived as helpful in previous EMA research (24), however, participants of the present study described it as only providing short-term relief that did not address the underlying drivers of the crisis. In a similar vein, substance use was described as reinforcing suicidal thoughts and reducing the capacity to inhibit suicidal urges, consistent with evidence showing that higher alcohol consumption and alcohol use prior to suicide attempts are associated with increased impulsive suicidal behavior (35, 36). This sequence of strategies was associated with increasing negative affect and suicidal thoughts and facilitated progression toward the justification of suicide, preparatory behaviors, and, in some cases, suicidal actions. In particular, suicide-related preparatory behaviors contributed to this escalation by providing a sense of control over an otherwise uncontrollable situation, while simultaneously restricting access to suicide-avoidant coping strategies and increasing proximity to suicidal action.

In this regard, suicidal behavior was not uniformly experienced as an intentional coping strategy. While some participants described suicide attempts as planned and goal-directed efforts to escape intense negative affective states, others reported these behaviors as occurring suddenly and without prior planning in a in state of acute loss of control and impulsivity, reflecting a breakdown of self-regulatory capacity rather than an intentional regulatory effort. This distinction is consistent with the assumptions of the DSMS (19), which posits that suicidal behavior may emerge either from goal-directed regulatory processes or from a breakdown of self-regulatory capacity, in which impulsive processes dominate as regulatory resources become depleted. In this context, persistent discrepancies between current and desired states, combined with diminished self-control, may increase the likelihood of unplanned suicidal behavior (37).

With regard to suicide-avoidant strategies, the commonly recommended strategy of disclosing suicidal thoughts was experienced as ambivalent, as its benefit strongly depended on the availability and reactions of others, which is consistent with previous findings (22). Participants frequently described concealing suicidal thoughts due to concerns about burdening others, internalized social norms, and fear of stigma, which discouraged self-disclosure and contributed to withdrawal and increasing social isolation. This is in line with meta-analytical evidence showing that between 50 and 60% of people who had experienced STBs had not disclosed them to anyone else (38). These interpersonal dynamics may further reduce access to potentially protective strategies and are consistent with findings indicating lower rates of disclosure among individuals who died by suicide (38).

### 4.3 Situational and contextual influences on coping

A central finding of the present study is the dependence of strategy selection and effectiveness on situational and contextual factors. Rather than being inherently adaptive or maladaptive, strategies appeared to vary in their effectiveness depending on the context in which they were applied. In particular, interpersonal conditions, such as the availability of supportive others and the quality of their responses, played a key role in determining whether coping efforts resulted in relief or further distress. Importantly, structural barriers such as long waiting times for psychotherapy or limited availability of low-threshold support services further restricted access to professional help, potentially fostering suicide-approach behaviors, which is consistent with previous research (18).

Furthermore, participants described emotional and physiological exhaustion resulting from prolonged stress due to various reasons (e.g., sleep disturbances, financial difficulties, or a non-supportive environment) as contributing to increasing distress and vulnerability during suicidal crises. Participants further reported that, during periods of heightened distress, they often relied more heavily on suicide-approach coping strategies, whereas more effortful strategies, such as seeking social or professional support or engaging in problem-focused coping, were perceived as increasingly difficult to access or implement. As a result, strategies typically considered adaptive were often described as unavailable due to environmental barriers or internal constraints. These recurring themes informed the participant-derived conceptual model presented in **Figure 1**.

Overall, these findings suggest that not all strategies typically considered adaptive are universally beneficial and that coping with suicidal thoughts and urges cannot be understood in a one-size-fits-all manner, as the applicability and effectiveness of strategies depend on highly heterogeneous situational and contextual conditions, including emotional and physiological distress, motivational dynamics, and social and structural contextual factors.

#### 4.3.1 The role of perceived self-control

In this context, perceived self-control played a pivotal role in shaping coping processes. Participants described self-control as a dynamic capacity that tended to decrease over the course of a suicidal crisis due to increasing emotional and physical exhaustion. In some situations, participants reported that the perceived availability of self-control enabled them to engage in safety-oriented strategies, such as delaying actions, or implementing safety strategies to interrupt suicidal behavior. While self-control is typically conceptualized as the capacity to inhibit undesired behavior, participants also described deliberately suppressing negative emotions, concealing suicidal thoughts, and maintaining functioning despite severe internal distress. These accounts suggest that self-control may also be directed toward the pursuit of maladaptive long-term goals, such as avoiding self-disclosure or maintaining an appearance of functioning despite ongoing psychological suffering. Importantly, these efforts were often perceived as cognitively demanding and exhausting. Participants further reported that sustaining such effortful control over extended periods reduced the availability of resources for alternative coping strategies and, in some cases, was followed by a perceived loss of control over suicidal thoughts and urges.

Taken together, participants’ accounts suggest that perceived self-control may fluctuate over the course of suicidal crises and is not necessarily experienced as adaptive. Participants described using self-control not only to inhibit suicidal urges but also to maintain maladaptive patterns, which they perceived as contributing to the persistence and escalation of STBs.

### 4.4 Clinical implications

The present findings have several implications for clinical practice. First, suicidality should not be conceptualized solely as the accumulation of risk factors, but rather as a dynamic and complex self-regulatory process that unfolds within an individual’s situational and contextual environment. This perspective highlights the importance of understanding how individuals regulate emotional distress and how contextual conditions shape the accessibility and effectiveness of coping strategies. Second, the findings underscore the central role of emotion regulation in the development of STBs. Effective intervention may require supporting individuals in recognizing and tolerating negative emotional states as a prerequisite for adaptive regulation. In line with the present results, many participants relied predominantly on avoidance-oriented strategies, which, although providing short-term relief, appeared to contribute to the escalation of distress over time. Accordingly, clinical interventions should focus on evaluating patients’ existing coping strategies and fostering a flexible, situation-sensitive repertoire of ERS. Third, the findings suggest that suicidal thoughts itself may serve a dysfunctional regulatory function by providing a sense of control, while simultaneously preventing engagement with underlying problems. Psychoeducation may therefore help patients to understand the short-term benefits and long-term costs of such strategies and to develop alternative coping approaches that target the underlying drivers of distress rather than providing only transient relief. Fourth, the effectiveness of commonly recommended strategies, such as self-disclosure, appears to depend on social and structural conditions. Clinicians should therefore carefully assess whether the social environment is supportive and whether adequate professional resources are accessible, as these factors may determine whether certain strategies are helpful or potentially harmful.

Finally, participants’ accounts suggested that perceived self-control may fluctuate over the course of a suicidal crisis and play an important role in coping with suicidal thoughts and urges. Interventions should not only aim to strengthen adaptive coping strategies but also to support the preservation and restoration of self-regulatory capacities. This may include reducing internal and external stressors, identifying maladaptive long-term goals that chronically deplete self-regulatory resources, improving sleep quality and physiological recovery, addressing substance use, and considering ongoing contextual burdens such as financial strain or caregiving responsibilities. Given that participants frequently described emotional exhaustion and diminished self-control during suicidal crises, interventions may also benefit from emphasizing coping strategies that require relatively few cognitive and self-regulatory resources. Such strategies may include brief mindfulness exercises, breathing techniques, sensory grounding exercises, structured safety plans, crisis cards, or other pre-planned coping routines that can be implemented with minimal cognitive effort. Developing and rehearsing these strategies in advance may increase their accessibility during periods of acute distress when more effortful forms of coping become difficult to implement. Taken together, these findings emphasize the need for individualized, context-sensitive interventions that address both regulatory processes and the conditions under which coping strategies are deployed.

### 4.5 Strengths and limitations

The present study has several notable strengths alongside methodological limitations. First, the use of a high-risk inpatient sample provided in-depth insights into coping processes in individuals with lived experience regarding suicidal crises, a population that is often underrepresented in research. Second, the qualitative approach allowed for a nuanced exploration of a broader range of ERS and SCS, as well as their subjective functions and temporal dynamics. At the same time, several limitations should be considered when interpreting the findings. Although the sample size (N = 12) was appropriate for qualitative research and allowed for thematic saturation, the results cannot be generalized to the broader population of individuals with STBs. Participants were recruited exclusively from inpatient psychiatric settings in Germany, excluding individuals without access to care, those who died by suicide, and individuals from other cultural or healthcare backgrounds. This may have led to a bias toward individuals who survived a crisis and were able to access treatment, potentially overestimating the use of suicide-avoidance coping strategies. Although transferability to other populations cannot be assumed, several identified themes, such as emotional overwhelm, avoidance-oriented coping, the perceived relief associated with suicidal thoughts, and the importance of social support, are consistent with findings from previous research on STBs (16). Future studies should examine whether these patterns are also evident in outpatient, community, and culturally diverse samples. In addition, the retrospective design introduces the possibility of recall bias, as participants reconstructed their experiences within an inpatient setting during or shortly after acute suicidal crises, potentially leading to a more coherent narrative and an overemphasis on helpful strategies. Moreover, retrospective self-reports were not triangulated with other data sources, such as ecological momentary assessment, behavioral measures, clinical records, or reports from significant others. Consequently, the identified processes reflect participants’ subjective reconstructions of their experiences and may not fully correspond to how these processes unfolded in real time. Furthermore, the interview guide explored suicide-specific coping strategies in relation to both suicidal thoughts and suicidal urges without systematically differentiating between these experiences. As participants often described suicidal thoughts and urges as closely intertwined, it was not always possible to determine whether specific coping strategies were primarily directed at suicidal thoughts, suicidal urges, or both. Future research may benefit from examining these processes separately. Furthermore, interviews conducted during or shortly after inpatient treatment may have been influenced by therapeutic interventions and psychoeducation, and social desirability bias may have led participants to emphasize socially acceptable strategies while downplaying more stigmatized behaviors. Finally, participants’ accounts may have been influenced by the interview context and the characteristics of the interviewers. The interviews were conducted by researchers with different levels of clinical and research experience in the field of suicidality, which may have influenced how participants presented their experiences and how certain topics were explored during the interviews.

## 5 Conclusion

The present study provides novel insights into emotion regulation and coping processes that characterize suicidal crises from the perspective of individuals with lived experience. The findings suggest that suicidal crises unfold as dynamic self-regulatory processes involving escalating emotional distress, changes in the accessibility and use of coping strategies, and fluctuating regulatory resources. Participants’ accounts indicate that while some strategies may provide temporary relief, prolonged reliance on effortful control and avoidance may deplete self-regulatory resources over time, thereby limiting access to suicide-avoidant coping and increasing vulnerability to suicide-approach processes and suicidal behavior.

These findings highlight the importance of understanding suicidal crises not only in terms of a specific constellation of risk factors, but as a dynamic process shaped by the interaction of emotional, motivational, and contextual influences. Moving beyond a primary focus on risk prediction, a process-oriented perspective may help to generate a more fine-grained understanding of how suicidal crises develop and unfold, thereby informing the development of more context-sensitive and tailored interventions that strengthen protective coping.

## Data Availability

The raw data supporting the conclusions of this article will be made available by the authors, without undue reservation.
