# Supplementary material for: From emotion regulation to suicide-specific coping: a qualitative study of self-regulatory processes in suicidal crises
Source: Front Psychiatry. 2026 Jul 24;17:1858456. doi: 10.3389/fpsyt.2026.1858456 (PMC13448552; doi:10.3389/fpsyt.2026.1858456)
Supplement: Supplementary file 1 [file SupplementaryFile1.docx]

**Interview Guide**

Today, I would like to conduct an interview with you to better understand your personal experience with your suicidal crisis. First, I would like to listen to your account of how this suicidal crisis or suicide attempt came about. After that, I would like to ask you a few questions.

Introduction suicidal narrative:

*Please tell me your story in your own words — how your suicidal crisis or suicide attempt came about…*

Structured questions:

**Suicide attempts**

1. For patients with acute suicidal thoughts but no recent suicide attempt: Have you ever attempted to take your own life?
2. How many suicide attempts have you undertaken?
3. How old were you at your first attempt?
4. When was your last suicide attempt?
5. Which method did you use?

**Trigger for the current suicidal crisis**

1. Were there certain triggers for your suicidal crisis?

**Emotions**

1. What triggers strong negative feelings in you?
2. What emotions cause you to have suicidal thoughts?
3. How do you cope with these emotions?
   - What helps you cope with distressing emotions?
   - What strategies cause your distressing emotions to intensify?

**Coping strategies for suicidal thoughts**

1. What triggers intense suicidal thoughts in you?
2. What do you do when you have (strong) suicidal thoughts? (internal vs. external strategies?)
3. What do you do most often?
4. What factors determine when you use which strategy (e.g., suicidal ambivalence, context, sleep problems, perceived burdensomeness)?
5. For patients with acute suicidal thoughts but no recent suicide attempt: What made you decide to be hospitalized because of your suicidal thoughts?

**Coping strategies for suicidal impulses and self-control**

1. What triggers your suicidal impulses?
2. What do you do when you experience suicidal impulses?
3. What strategies increase your suicidal impulses?
4. What strategies do you use most often?
5. What determines whether or not you can control the impulse and prevent yourself from attempting suicide (suicidal ambivalence)?
6. How can you tell that your self-control is diminishing and you can barely stop yourself from taking your own life?
7. Only for patients with a suicide attempt:

How did you deal with the suicidal impulse during your last suicide attempt? In your

opinion, what led to you being unable to stop yourself from attempting suicide?

Why did you attempt suicide on that particular day and not on another day?

**Resource-oriented questions**

1. How can you tell that your suicidal thoughts are going away?
2. Are there any strategies that help you reduce suicidal thoughts and distance yourself from the issue of suicidality?
3. What would help you have fewer suicidal thoughts?
4. Do you talk to others about your suicidal thoughts? If so, does it help?
5. What would help you have fewer suicidal impulses?
6. Are there any strategies you learned during your hospital stay?

**Sleep problems**

1. Do you suffer from sleep problems?
2. If so, which?
3. Does your sleep affect how you deal with suicidal thoughts or your suicidal impulses?

**Substance use**

1. Do you drink alcohol or use other substances?
2. Does the use of alcohol or other substances affect how you deal with suicidal thoughts or suicidal impulses?
