# Supplementary material for: From emotion regulation to suicide-specific coping: a qualitative study of self-regulatory processes in suicidal crises
Source: Front Psychiatry. 2026 Jul 24;17:1858456. doi: 10.3389/fpsyt.2026.1858456 (PMC13448552; doi:10.3389/fpsyt.2026.1858456)
Supplement: Supplementary file 2 [file Table1.docx]

Supplemental Table S1: Example Quotes of Emotion Regulation Strategies (ERS)

| Strategy Category | Description | Example Quote |
| --- | --- | --- |
| **Avoidance-oriented ERS** |  |  |
| Suicidal ideation | Engaging with thoughts of ending one’s own life as a strategy to regulate emotional distress, providing short-term relief and a perceived sense of escape or way out. | *“With the self-hatred and the sadness, suicidal thoughts felt like a kind of mental escape. Somehow, it felt like everything was hopeless, but at the same time, there was still this kind of emergency exit.”* (int 04) |
| Suppression | Intentional suppression or concealment of emotions to avoid outward expression of negative emotions. | *“I kept functioning and didn’t want to acknowledge that I was slipping into depression. I started working, but after about a month, I became so overwhelmed that I couldn’t function anymore.”* (int 05) |
| Self-devaluation | Participants described themselves as highly self-critical and self-devaluing. | *“I feel disappointed that I can’t manage my life the way I had always imagined. Things used to come easily, but now everything feels extremely difficult and it somehow doesn’t works out the way I want it to. Because of that, I don’t like myself anymore—I’m not just dissatisfied, I’m actually angry with myself.”* (int 04) |
| Rumination | Repetitive, ruminative thinking about problems or the causes of the crisis. | *“I was so stuck in this cycle of thoughts about the separation from my husband (…) that I eventually became completely overwhelmed.”* (int 05) |
| Withdrawal | Withdrawal from social contacts or everyday activities to protect oneself from emotional overwhelm or from confrontation with others. | *“When I feel extremely empty inside, I notice that I start to withdraw again.”* (int 11) |
| Substance use | Use of alcohol, medication, or drugs to reduce the intensity of negative affective states. | *“I often smoked cannabis before work - just to be able to endure being there.”* (int 02) |
| Self-injury | Deliberate self-inflicted physical harm used to regulate affective states (e.g., to reduce tension). | *“It has happened that I started harming myself again, because there was this feeling of shame - this sense of being worthless - and this inner pain. I tried to numb that by replacing it with physical pain.”* (int11) |
| Distraction | Shifting attention to other activities to temporarily distance oneself from distressing emotions. | *“I also spent a lot of time on the console or computer to distract myself - to sort of enter a different reality.”* (int02) |
| Escape | Leaving or actively avoiding distressing situations, with a primary focus on immediately withdrawing from contexts perceived as uncontrollable, overwhelming, or threatening. | *“It was the same with all the doctor’s appointments here. At one point, I even ran off because I had a panic attack - because I couldn’t control what would happen next.”*  (int 01) |
| **Approach-oriented ERS** |  |  |
| Self-disclosure | Verbal or written communication of one’s emotions and internal states to others to seek understanding, validation, or relief. | *“Nothing was working anymore. I felt like I was doing everything wrong. So, I went to my boss’s office and, for the first time, broke my silence and said, ‘I don’t know what’s going on with me.’ At that point, I didn’t really know anything about this illness or what was happening to me.”* (int 11) |
| Seeking support | Sharing thoughts and emotions with others (e.g., friends, family, professionals) to seek support and reduce emotional burden. | “At one point, I felt so bad that I texted her, saying I wasn’t doing well and that I needed her and wanted to talk on the phone.” (int02) |
| Confrontation | Engaging with and allowing distressing emotions in order to reduce their intensity or to cope with them more effectively in the long term.” | *“I consciously face the anxiety and try to endure it.”*  (int 01) |
| Emotional expression | Open expression of emotions (e.g., crying, shouting, expressing anger) to reduce internal tension. | *“I even had to cry, and she would always say, ‘Don’t cry!’ And I said, ‘Are you crazy? I’m glad I can do it again - the doctor said it has to come out. For me, it’s a relief.’”*  (int 05) |
| Cognitive reappraisal | Reinterpreting a situation to change its emotional impact. | *“I mean, I didn’t necessarily do the two years of training as a social care assistant in vain. I was able to gain a lot of experience and develop many values, but at first it was difficult for me to recognize that.”* (int 02) |
| Activation | Deliberate activities aimed at regulating negative affect and tension, which either have a preventive stabilizing effect or provide short-term relief during acute distress. | *“I really enjoy doing crafts. For me, it’s a great emotional balance, and I make use of that during those times.”*  (int 08) |
