# Supplementary material for: From emotion regulation to suicide-specific coping: a qualitative study of self-regulatory processes in suicidal crises
Source: Front Psychiatry. 2026 Jul 24;17:1858456. doi: 10.3389/fpsyt.2026.1858456 (PMC13448552; doi:10.3389/fpsyt.2026.1858456)
Supplement: Supplementary file 3 [file Table2.docx]

Supplemental Table S2: Example Quotes of Suicide-Coping Strategies (SCS)

| Strategy Category | Description | Example Quote |
| --- | --- | --- |
| **Approach-oriented SCS** |  |  |
| Justifying suicide | Perceiving one’s life as meaningless or lacking perspective, and identifying this as a reason for attempting suicide. | *“I keep falling back into this hole again: ‘What if I just wasn’t here anymore? Then I wouldn’t have to go through this therapy. Then I’d be free. Then I could finally decide for myself and be the way I want to be, instead of letting others decide for me.’”* (int 03) |
| Suicide-related preparatory behavior | Engaging in behaviors aimed at preparing for a potential suicide attempt. | *“It escalated to the point that I began planning an attempt. I wrote out a plan and had already started working through the list.”* (int 09) |
| Withdrawal | Suicide-related withdrawal from social contacts or daily activities, motivated by suicidal intent and aimed at preventing intervention by others. | *“I rejected the help—I pushed away the people who cared about me and wanted to support me, and I felt as if I was in a kind of tunnel.”* (int 10) |
| Distraction | Engaging in behaviors to temporarily distract from acute suicidal thoughts or urges in order to prevent suicidal behavior. | *“At home, I try to get through it by watching TV or a series until I eventually fall asleep […].”* (int 12) |
| Suppression | Avoiding or suppressing suicidal thoughts to prevent disclosure and avoid receiving help. | *“I met a friend, and she said, ‘Something’s off with you. Should I drive you home? Give me your car keys.’ But I said, ‘No, it’s fine.’, and refused it”* (int 10) |
| Substance use | Use of alcohol, medication, or drugs to temporarily reduce the intensity of suicidal thoughts or urges. | *“I frequently used cannabis, often when I was on my own. It initially suppressed the thoughts, but they returned even stronger once the effects wore off.”* (int 02) |
| Suicidal behavior | Deliberate self-harming behaviors with the intent to die. | *“I was sitting on the chair, and after I had made the twelve cuts, the blood was dripping, of course, through my trousers and shirt. […] I don’t know how much blood had already come out of my body.”* (int 06) |
| **Avoidance-oriented SCS** |  |  |
| Professional support | Use of professional services in response to suicidal ideation or urges (e.g., psychotherapy, inpatient care, crisis services). | *“I approached the doctor and said, ‘Doctor, I heard you talking about the psychiatric clinic, and I definitely need help. I would like to admit myself there and receive treatment.’”* (int 06) |
| Social support | Use of social support in response to a suicidal crisis (e.g., assistance with tasks or decisions) | “And then I told him that I hoped I would manage, and he said, ‘Mom, we’ll support you - we’ll help you.’ That gave me the strength and the hope to see something positive again.” (int 05) |
| Disclosing suicidal ideation | Communicating suicidal thoughts, urges, or behaviors to others to make one’s situation known and seek emotional support. | *“I eventually told her about my suicidal thoughts and my suicide attempt.”* (int 02) |
| Focusing on reasons for living | Reflecting on the meaning or value of one’s life to counteract suicidal thoughts (e.g., through personal values, beliefs, or existential reflection). | *“I remind myself what makes life worth living—what I’ve achieved, how far I’ve come, and that I have a son and a family. That’s what holds me back, even though the thoughts are still there.”* (int 10) |
| Questioning the desire to die | Questioning suicidal intent, and reflecting on the potential consequences of a suicide attempt. | *“Another important thought was: how would I leave people behind? That was a major consideration influencing my suicide plan—how my mother would react, how my friends would react, how my case worker would react.”* (int 09) |
| Self-soothing | Behaviors aimed at self-soothing and encouraging oneself in the context of a suicidal crisis, with the goal of restoring emotional control. | *“When they left and I was alone again, I thought to myself, ‘You have to be strong now. Set yourself something to do for the day, think about what you’ll do after the clinic, and take it step by step—it will work out.’”* (int 05) |
| Problem identification | Identifying key problems or stressors underlying STBs. | *“And then I said, ‘Listen, I can do 30 weeks of therapy here, but if nothing changes at home - if you don’t change - even the best therapy won’t help me.’”* (int 12) |
| Future planning | Actively modifying distressing external circumstances to reduce stressors related to STBs and facilitate long-term relief and stabilization. | *“I decided for myself that I needed a complete fresh start.”* (int 06) |
| Other safety strategies | Actions taken by the individual or close others to prevent suicidal behavior (e.g., use of a safety plan). | *“At some point, I had to stop along the way because I couldn’t go on. I got out of the car and just left it there.”* (int 10) |
| Emergency contacts | Engaging emergency or crisis services or presenting to acute clinical care settings (e.g., calling emergency services or a pre-identified contact person). | *“At one point, I called someone who sent an ambulance, which arrived just in time.”* (int 01) |
